# Repeatability of in vivo quantification of atherosclerotic carotid artery plaque components by supervised multispectral classification

**DOI:** 10.1007/s10334-015-0495-2

**Published:** 2015-07-11

**Authors:** Shan Gao, Ronald van ’t Klooster, Diederik F. van Wijk, Aart J. Nederveen, Boudewijn P. F. Lelieveldt, Rob J. van der Geest

**Affiliations:** Division of Image Processing, Department of Radiology, Leiden University Medical Center, P.O. Box 9600, 2300 RC Leiden, The Netherlands; Department of Vascular Medicine, Academic Medical Center, Amsterdam, The Netherlands; Department of Radiology, Academic Medical Center, Amsterdam, The Netherlands

**Keywords:** Cardiovascular disease, Carotid artery, Atherosclerotic plaque, Multi-contrast MRI, Classification, Repeatability

## Abstract

**Objective:**

To evaluate the agreement and scan–rescan repeatability of automated and manual plaque segmentation for the quantification of in vivo carotid artery plaque components from multi-contrast MRI.

**Materials and methods:**

Twenty-three patients with 30–70 % stenosis underwent two 3T MR carotid vessel wall exams within a 1 month interval. T1w, T2w, PDw and TOF images were acquired around the region of maximum vessel narrowing. Manual delineation of the vessel wall and plaque components (lipid, calcification, loose matrix) by an experienced observer provided the reference standard for training and evaluation of an automated plaque classifier. Areas of different plaque components and fibrous tissue were quantified and compared between segmentation methods and scan sessions.

**Results:**

In total, 304 slices from 23 patients were included in the segmentation experiment, in which 144 aligned slice pairs were available for repeatability analysis. The correlation between manual and automated segmented areas was 0.35 for lipid, 0.66 for calcification, 0.50 for loose matrix and 0.82 for fibrous tissue. For the comparison between scan sessions, the coefficient of repeatability of area measurement obtained by automated segmentation was lower than by manual delineation for lipid (9.9 vs. 17.1 mm^2^), loose matrix (13.8 vs. 21.2 mm^2^) and fibrous tissue (24.6 vs. 35.0 mm^2^), and was similar for calcification (20.0 vs. 17.6 mm^2^).

**Conclusion:**

Application of an automated classifier for segmentation of carotid vessel wall plaque components from in vivo MRI results in improved scan–rescan repeatability compared to manual analysis.

## Introduction

Multi-contrast magnetic resonance imaging (MRI) has demonstrated the capability of in vivo characterization of the morphological (area, volume) [1, 2] and compositional (lipid, calcification, loose matrix, intra-plaque hemorrhage) [2–5] features of human carotid atherosclerotic plaque. Therefore, longitudinal carotid MRI could potentially provide unique insight into possible morphological changes of plaque components due to disease progression, or the effect of medical treatment. The assessment of changes in the component volume over time is affected by the accuracy and the repeatability of the image acquisition and the subsequent image analysis.

Acceptable intra-observer and inter-observer reproducibility for identifying and quantifying carotid plaque components has been demonstrated in in vivo MRI experiments [6]. Recent studies have evaluated the repeatability of plaque composition assessment based on scan–rescan imaging using 1.5 T and 3T MRI [7, 8]. However, these studies heavily relied on manual segmentation, which is a labour-intensive and time-consuming procedure that becomes impractical for studies that require the segmentation of many carotid plaques. Several studies have evaluated the feasibility of automated techniques for in vivo MR plaque segmentation [9–13]. These studies have demonstrated promising results, as indicated by the good agreement with manual plaque segmentation [9, 12] or histological findings [10, 11, 13]. However, very few studies have assessed the inter-scan repeatability of automated plaque classification. To our knowledge, only one study [14] reported on this aspect based on 1.5T MRI data. While carotid imaging at 3T has demonstrated improved signal-to-noise (SNR) compared to 1.5T [15], no studies have investigated the repeatability of automated plaque segmentation on 3T.

Accordingly, the purpose of this study was to assess the scan–rescan repeatability of an automated supervised classification system and manual segmentation for the quantification of atherosclerotic plaque components from multi-contrast carotid vessel wall MRI at 3T. In addition, we assessed the agreement between automated classification and manual segmentation by an experienced reader.

## Materials and methods

### Study population

Thirty-one patients, with one or more atherosclerotic events (stroke and/or myocardial infarction) and 30–70 % stenosis in one of the carotid arteries, identified by duplex ultrasound measurement, were scheduled for a first and a second carotid vessel wall MR exam within a 1-month interval. Two patients cancelled their second appointment. Three other patients did not complete their MRI exams, due to claustrophobia and severe discomfort. After exclusion, 26 patients participated in this study and no major clinical events were reported in the period between the two exams. The image quality (ImQ) was evaluated by an experienced reader and graded using a 4-point scale (grade 1 = poor, grade 4 = excellent) based on the overall SNR and motion artifacts. Images of three patients with poor quality (ImQ = grade 1) were excluded because the carotid wall and vessel boundaries were unidentifiable. The remaining 23 patients’ first and second scans, constituting 46 data sets, were used in this study. Baseline characteristics of the patient population are given in Table 1.

**Table 1:** Baseline characteristics of the patient population

	Mean ± SD or % (ratio)
Patient characteristics (*n* = 31)	
Age (years)	68.8 ± 7.5
Women (%)	48 % (15/31)
Body mass index (kg/m^2^)	25.4 ± 2.9
Ultrasound dimensions	
IMT_CC_ (mm)	1.10 ± 0.56
IMT_BULB_ (mm)	1.73 ± 0.72
IMTI_CA_ (mm)	1.02 ± 0.56
MRI dimensions	
Total wall volume (mm^3^)	735.0 ± 431.8
Mean wall area (mm^2^)	53.0 ± 29.6
Mean wall thickness (mm)	2.07 ± 0.63

*IMT*_*CC*_ intima-media thickness of common carotid, *IMT*_*BULB*_ intima-media thickness of carotid bulb, *IMTI*_*CA*_ intima-media thickness of internal carotid artery

### Carotid MRI

All MRI examinations were performed on a 3T whole body MR scanner (Intera, Phillips Healthcare, Best, The Netherlands) using an eight-channel bilateral carotid artery coil. Details of the imaging protocol have been described previously [16]. In short, axial angiography images acquired with a time of flight (TOF) sequence covering both carotid arteries (FOV = 10 × 10 cm^2^, 40 slices of 2 mm thickness, a segment of 8 cm was scanned), together with ultrasound duplex data, were used for planning the acquisition of multi-contrast vessel wall images. Subsequently ECG-gated axial TOF, PDw, T1w, T2w images were acquired with the acquisition stack positioned centered at the atherosclerotic plaque of the carotid artery, in which the plaque burden was most profound according to the ultrasound result. Overview images showing the image stack superimposed over the carotid artery were saved from the first scan for planning the acquisition of the second scan. The FOV (60 × 60 mm^2^), acquisition matrix (120 × 120), non-interpolated pixel size (0.5 × 0.5 mm^2^), number of slices (8), slice thickness (2 mm) and slice gap (0 mm) were identical for all four sequences. The black-blood T1w, T2w, and PDw images were acquired with an identical flip angle (90°). The repetition time was 2 R–R intervals for T2w and PDw, 1 R–R interval for T1w; and echo time was 8 ms for T1w and PDw and 50 ms for T2w. The TOF sequence used the following parameters: flip angle 20°; echo time 5 ms; repetition time 19 ms. To remove the effect of inhomogeneous sensitivity of the carotid surface coil, constant level appearance (CLEAR) image reconstruction was applied.

### Manual image review

Images of the 46 exams were examined by an experienced MRI reader, who was fully blinded to the scan session and patient information. To assess the intra-observer agreement, the reader, blinded to the previous annotation result, reanalyzed all the blinded images 2 months after the initial review. The manual segmentation result of the initial review is further denoted as *first read* and the results of the second review as *second read* in the remainder of this manuscript. Image sets of each patient obtained from scan and rescan sessions were randomized and anonymized to prevent any recall bias when performing repeated review of the same patient. Image review and manual analysis were performed using VesselMass software (Leiden University Medical Center, the Netherlands) [9, 17]. During the procedure, four contrast-weighted images of a given slice were simultaneously presented. Next, lumen and outer wall boundaries of the common and the internal carotid artery were manually traced on the T1w image and propagated to the images of other weightings. T2w, PDw and TOF images were manually registered to the T1w image by translating each image stack in the through-plane direction and each slice in the in-plane direction to match the contours of the inner and outer wall, such that patient motion between acquisitions was corrected, resulting in an aligned set of multi-contrast images. Regions of lipid, calcification, ulceration, hemorrhage and loose matrix were delineated according to previously described and validated plaque classification criteria [2, 3, 18, 19], and were based on relative intensities observed in the four sequences, such as lower, higher, or equal to adjacent sternocleidomastoid (SCM) muscle.

To enable assessment of the scan–rescan repeatability of plaque classification at a slice level, registration between images of the scan and rescan sessions was performed. The alignment of the transverse slices in the repeat scans was performed manually using the T1w series. To this end, the second scan was aligned to the first scan by shifting the image stack in the through plane direction such that the bifurcation slice, which was defined as the image at the location crossing or just distal to the flow divider, corresponded to the bifurcation slice in the first scan.

### Automated plaque classification

The classifier for the automated plaque segmentation was constructed based on a previously described and validated method [12]. In brief, a supervised pattern recognition system was trained using the available manual segmentation results to automatically classify the plaque contents by using the intensity and morphological features extracted from multi-contrast MRI. For the 23 patients, a total of 46 data sets were included in the automated analysis. Sixty-four MRI slices were excluded because after inter-sequence registration not all contrast weightings were available, which resulted in 7 ± 1 (range 3–8) MRI slices per data set for which information from all four sequences (T1w, T2w, PDw, TOF) was available. To allow the comparison of signal intensity (SI) between different images, intensity normalization was performed slice by slice, in which MR images of all contrast weightings were divided by the median SI of a 4 cm diameter circular ROI centered at the lumen. In each slice, the pixels in the carotid artery vessel wall, as defined by the lumen and outer wall boundaries, were extracted to create data sets. For each pixel, the following features were calculated: normalized SI, zero-order, first-order and second-order derivatives at five scales (*σ* = 0.1, 0.25, 0.5, 1, 2 mm) of the four sequences, distance to the vessel lumen, distance to the outer wall and local wall thickness. A linear discriminant classifier (LDC) that models each class as a multivariate Gaussian distribution with an equal covariance matrix was trained based on the above intensity and morphological features. The classifier was evaluated by using a leave-one-patient-out cross-validation approach, in which all pixel samples in one data set from the first or second scan of one patient were used for testing, and all pixel samples in the first and second scans of the remaining 22 patients (44 data sets) were used for training. The segmentation provided by the first read was used to train the classifier; the reference standard was set by the same read. To remove the estimation bias of fibrous tissue volume between the observer and the automated classifier, the prior probability of fibrous tissue was set to 0.55. Next, the prior probabilities of plaque tissues were calculated according to the plaque component distribution in the training set, taking into account that the sum of prior probabilities should be equal to one. Each vessel wall pixel was classified to be one of the six classes: fibrous tissue, lipid, calcification, ulceration, hemorrhage, and loose matrix according to the highest posterior probability. A post-processing step was implemented to eliminate isolated pixels. Isolated pixels were relabelled to the majority class of the neighboring pixels (within a 3 × 3 pixels window). A pixel was considered to be isolated if it was the only pixel of a given plaque component in a slice. The automated classification experiments were performed using MATLAB 2011b (Mathworks, Natick, US) and the pattern recognition toolbox PRTools (version 4.2.0) [20].

### Qualitative and quantitative plaque component analysis

For each patient, the presence or absence of individual plaque component was assessed in the scan and rescan sessions. The absolute area of the detected plaque components (lipid, calcification, loose matrix), fibrous tissue and vessel wall was computed in each slice for statistical analysis. The absolute volume and the volume difference between scan and rescan were computed for each component and for the vessel wall from each patient. Due to the small number of cases in the patient population with ulceration or hemorrhage tissue, no further quantitative analysis was performed for these two plaque components.

### Statistical analysis

To compare the measurements between scan sessions (inter-scan), the Wilcoxon matched pairs test was performed as the differences between each pair of areas were not normally distributed, which was validated by the D’Agostino-Pearson normality test. To assess the scan–rescan repeatability of manual and automated plaque segmentation, Spearman’s correlation coefficient (Spearman’s *r*) and Bland–Altman plots [21] were calculated from the paired plaque areas obtained in the repeated scans of all 23 patients for each individual tissue type. Spearman’s *r* was used, as the distribution of the plaque region areas is non-Gaussian. According to Rosenstock et al. [22], Spearman’s *r* values between 0.8 and 1 indicate excellent agreement; 0.61–0.80 good agreement; 0.41–0.60 satisfactory agreement; 0.21–0.40 fair agreement; and below 0.20 poor agreement. According to the approach of Bland and Altman [21], the coefficient of repeatability (CR) is computed as two times the standard deviation (SD) of the paired inter-scan area differences if the bias between inter-scan measurements is close to zero, resulting in a low CR value for a segmentation method with high repeatability. To compare the SD of inter-scan area difference as well as the CR between manual and automated analysis, Levene’s test [23] was applied.

As the automatic plaque segmentation makes use of the manually delineated vessel wall region, Bland–Altman analysis was also performed for the paired vessel wall areas of all 23 patients to evaluate the repeatability of manual vessel wall segmentation.

The agreement between automated and manual segmentation was assessed using Spearman’s *r*. For all statistical tests, a *p* value <0.05 was considered to be statistically significant.

## Results

**Fig. 1:**
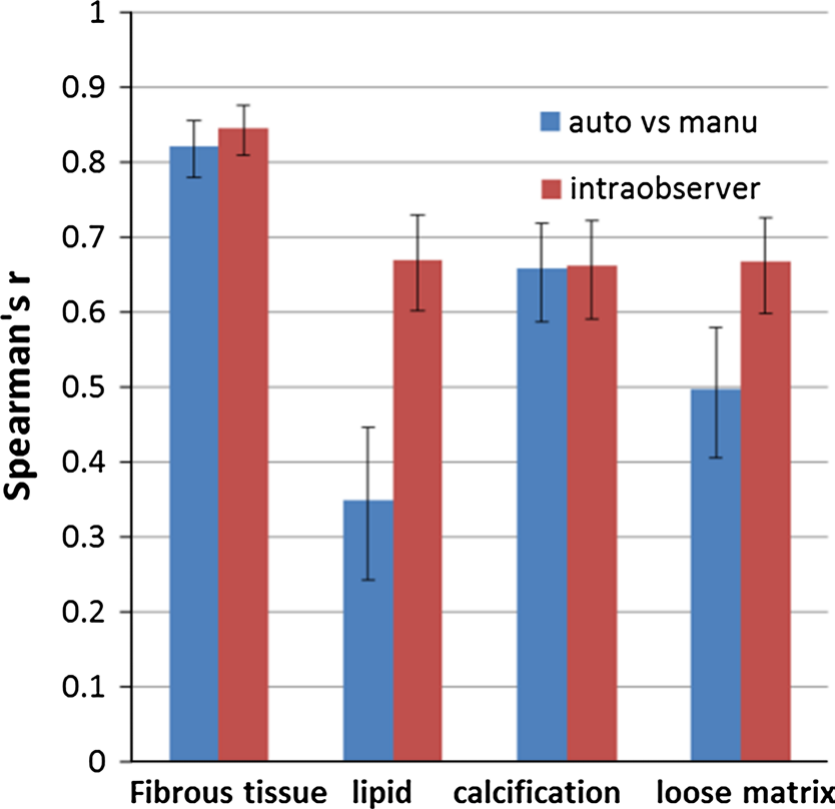
Comparison of agreement between automated and manual plaque classification with intra-observer agreement for manual analysis. The intra-observer agreement (*red bar*) is higher than the agreement between automated and manual segmentation (*blue bar*) for lipid and loose matrix, while it is similar for fibrous tissue and calcification. The *error bars* indicate the 95 % CI

**Fig. 2:**
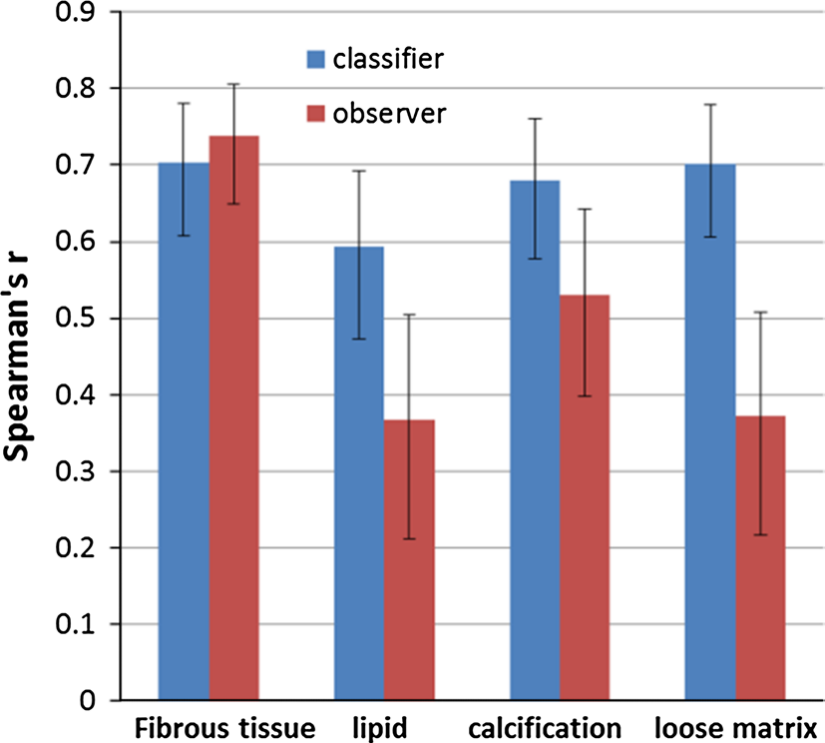
Repeatability of inter-scan quantitative assessment of plaque component areas obtained by automated and manual segmentation. The automated classifier (*blue*) demonstrates higher scan–rescan repeatability for plaque composition quantification compared to the observer (*red*). The *error bars* indicate the 95 % CI

**Fig. 3:**
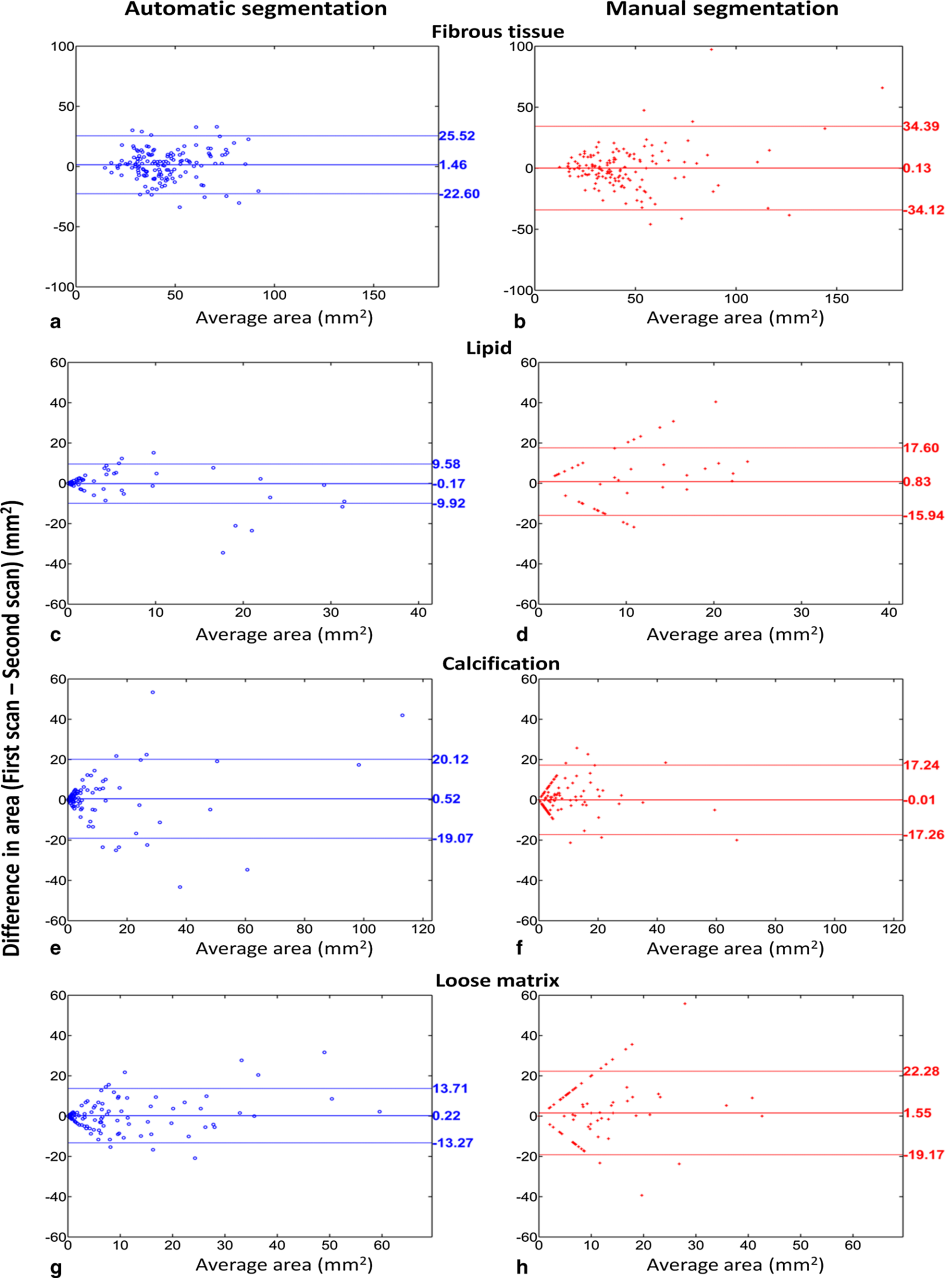
Bland–Altman plots for inter-scan plaque components area assessment. Compared to manual segmentation (*red*), automated segmentation (*blue*) shows more consistent detection of plaque components, indicated by less data points along a 63.5-degree line. The *middle horizontal line* indicates the bias and the *upper* and *lower horizontal lines* indicate the 95 % limits of agreement

**Fig. 4:**
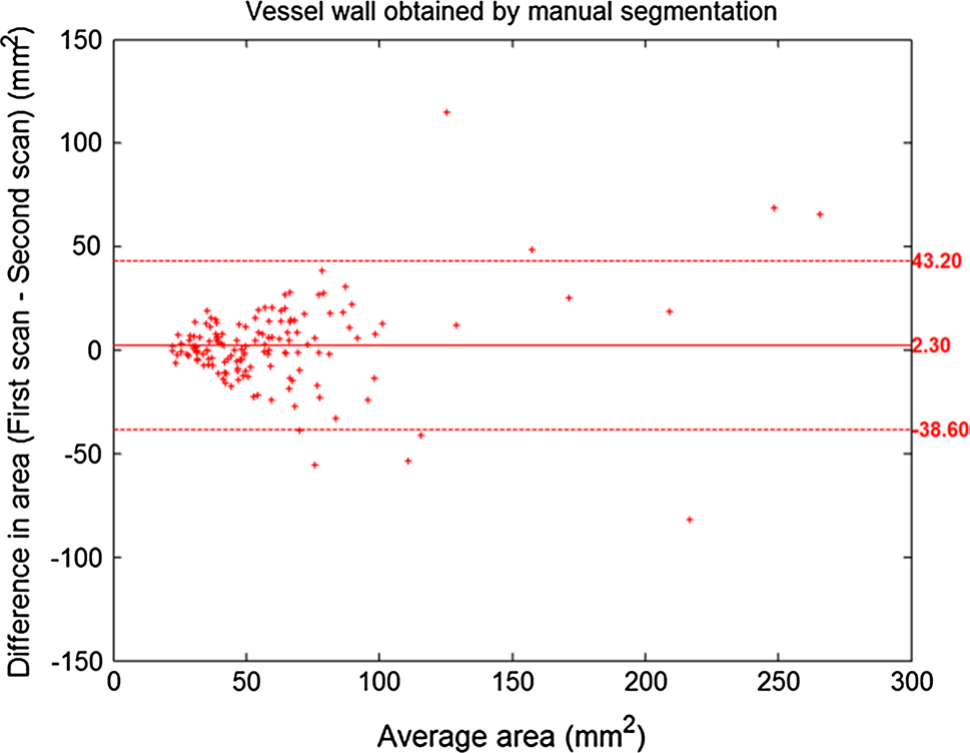
Bland–Altman plot for inter-scan vessel wall area assessment based on manually traced contours. The *middle horizontal line* indicates the bias and the *upper* and *lower horizontal lines* indicate the 95 % limits of agreement. The bias is not significantly different from 0 (*p* = 0.08), indicating the absence of significant bias between the quantified inter-scan vessel wall areas

Table 2 summarizes the baseline characteristics of the volume of particular tissue types and complete vessel wall from the pre-aligned first scan data set based on the first read. In total, 304 slices from unilateral carotid arteries (*n* = 46) from 23 patients were included in the segmentation experiment, in which 144 aligned slice pairs were available for repeatability analysis. The results of automated and manual atherosclerotic plaque segmentation are presented in Tables 3 and 4 and Figs. 1, 2, 3 and 4.

**Table 2:** Baseline characteristics of manually segmented fibrous tissue, plaque components and vessel wall

	Presence of plaque component	Volume (mm^3^) mean ± SD	Area (mm^2^) mean ± SD
Fibrous tissue	100 % (23/23)	598 ± 327	43.8 ± 26.9
Lipid	43 % (10/23)	108 ± 81	17.5 ± 10.3
Calcification	87 % (20/23)	98 ± 125	12.2 ± 11.7
Loose matrix	65 % (15/23)	133 ± 99	15.8 ± 10.8
Ulceration	26 % (6/23)	35 ± 71	8.7 ± 6.4
Hemorrhage	4 % (1/23)	18	4.6 ± 0.6
Vessel wall	100 % (23/23)	827 ± 541	60.6 ± 41.4

For a given component, the mean volume/area was calculated from the pre-aligned first scan data by taking the average from the patients/slices in which a certain component was identified

**Table 3:** Presence/absence of each plaque component in two scan sessions per patient (*n* = 23)

Plaque components	Manual segmentation			Automatic segmentation		
	Second scan			Second scan		
	First scan	Absence	Presence	First scan	Absence	Presence
Fibrous tissue	Absence	0	0	Absence	0	0
	Presence	0	23	Presence	0	23
Lipid	Absence	10	3	Absence	5	2
	Presence	4	6	Presence	5	11
Calcification	Absence	0	4	Absence	0	1
	Presence	2	17	Presence	4	18
Loose matrix	Absence	5	3	Absence	3	1
	Presence	5	10	Presence	2	17

**Table 4:** Volume measurement of each plaque component and vessel wall at scan–rescan sessions

Plaque component	Volume measured with manual segmentation (mean ± SD)			Volume measured with automatic segmentation (mean ± SD)		
	First scan (1st)	Second scan (2nd)	Difference (1st–2nd)	First scan (1st)	Second scan (2nd)	Difference (1st–2nd)
Fibrous tissue (mm^3^)	562.2 ± 354.5	560.6 ± 351.2	1.7 ± 108.0	569.6 ± 263.3	551.4 ± 265.2	18.3 ± 100.8
Lipid (mm^3^)	44.1 ± 75.1	33.7 ± 8.9	10.4 ± 64.8	28.6 ± 64.1	30.7 ± 83.2	−2.1 ± 26.1
Calcification (mm^3^)	82.9 ± 121.8	83.1 ± 145.2	−0.2 ± 55.3	85.4 ± 189.6	78.8 ± 175.2	6.6 ± 65.7
Loose matrix (mm^3^)	80.5 ± 101.4	61.1 ± 90.4	19.4 ± 104.3	86.2 ± 132.2	83.4 ± 110.9	2.8 ± 61.1
Vessel wall (mm^3^)	779.7 ± 570.4	750.9 ± 521.5	28.8 ± 161.9			

For each type of tissue, the mean volume was calculated by taking the average from all 23 patients, in whom the post-aligned scan and rescan data were used

### Comparison of plaque composition quantification between automated and manual segmentation

Figure 1 shows the comparison of the segmentation from automated and manual methods. For fibrous tissue and calcification, the agreement between the automated and manual analysis was similar to the intra-observer agreement of the experienced reader. For lipid and loose matrix, the agreement of repeated manual delineations was significantly higher than the automated-manual agreement, which is indicated by the non-overlapping confidence intervals (CI).

### Comparison of plaque component detection repeatability between automated and manual segmentation

An overview of inter-scan plaque component detection based on both segmentation methods is provided in Table 3. Both methods performed similarly in detecting calcification at the patient level. Compared to manual segmentation, the presence of lipid (11 vs. 6) and loose matrix (17 vs. 10) was consistently identified in more patients, and the presence of loose matrix (3 vs. 8) was inconsistently identified by automated segmentation in less patients.

### Comparison of plaque composition quantification repeatability between automated and manual segmentation

Figure 2 shows the scan–rescan repeatability of plaque area quantification using automated and manual segmentation expressed as the Spearman’s *r*. Results of the automated segmentation showed an increase in repeatability for most plaque components compared to the manual segmentation. The repeatability remained good for fibrous tissue, increased from fair to satisfactory for lipid, increased from satisfactory to good for calcification, and increased from fair to good for loose matrix.

The results of Bland–Altman repeatability analysis are shown in Fig. 3. For all plaque components, the bias was found to be not significantly different from zero. For both automated and manual segmentation, no significant difference was observed between scan and rescan areas for fibrous tissue (*p* = 0.10 and *p* = 0.88), lipid (*p* = 0.26 and *p* = 0.45), calcification (*p* = 0.11 and *p* = 0.84) and loose matrix (*p* = 0.70 and *p* = 0.09). Data points along a 63.5-degree line correspond to the locations in which a given plaque component was detected in one scan only. Inconsistent detection of plaque components, in which lipid and loose matrix were identified in the first (second) scan slice but not in the corresponding second (first) scan slice, was found more often in manual analysis. Compared to manual segmentation, repeatability of inter-scan area measurement was significantly higher for automated segmentation for lipid (CR: 9.9 vs. 17.1 mm^2^, *p* = 0.02) and loose matrix (CR: 13.8 vs. 21.2 mm^2^, *p* = 0.03); and was similar for calcification (CR: 20.0 vs. 17.6 mm^2^, *p* = 0.62) and fibrous tissue (CR: 24.6 vs. 35.0 mm^2^, *p* = 0.05).

The result of Bland–Altman analysis for comparing the inter-scan vessel wall areas obtained with manual contouring is presented in Fig. 4. No significant bias and no significant correlation between the bias and the size of vessel wall was found, suggesting the vessel wall segmentation performed by human observer was repeatable.

Table 4 summarizes the volumes and inter-scan volume difference of each plaque component detected by both segmentation methods. In addition, the vessel wall volumes measured at the two scan sessions based on manually traced lumen/outer contours are also listed in Table 4. Overall, the manual segmentation measured larger lipid volume and smaller loose matrix volume, while automated segmentation measured smaller inter-scan volume difference on these two plaque components.

**Fig. 5:**
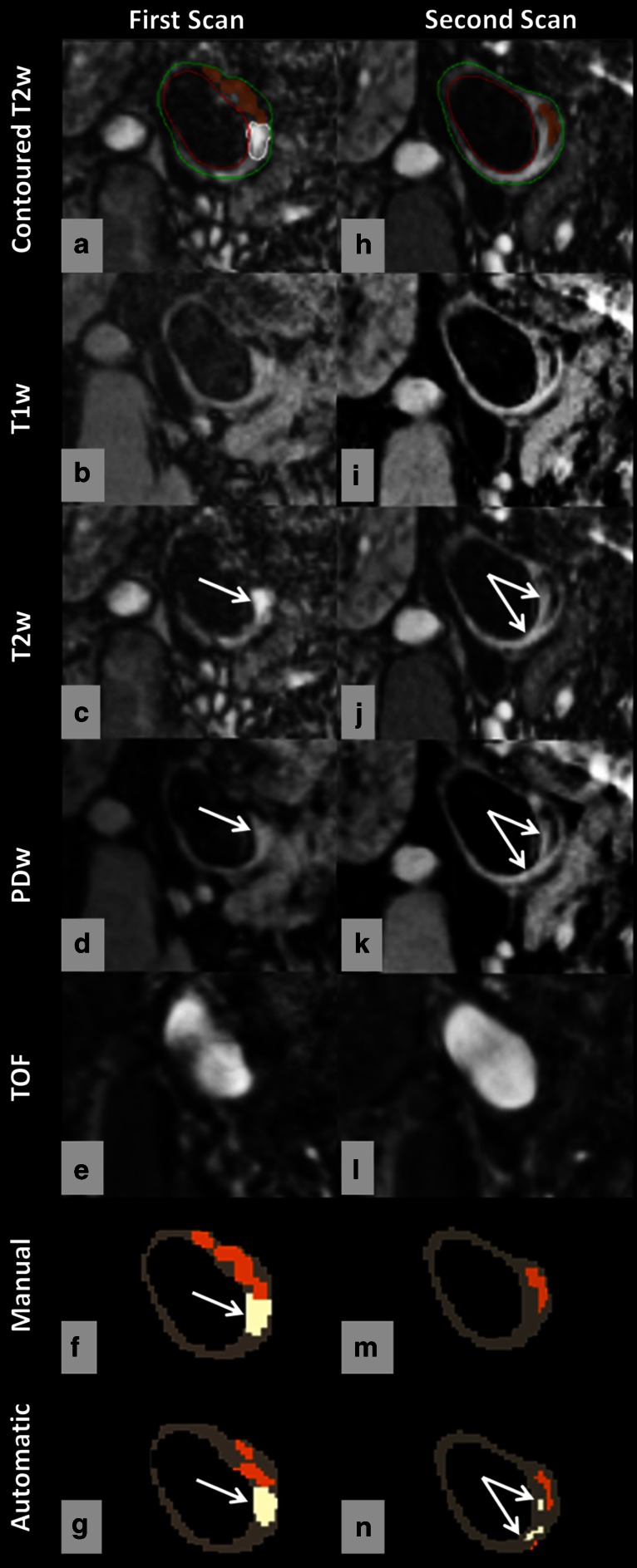
Manual and automated classification of loose matrix and calcification using multicontrast MR images, obtained in repeated scans. Manual contours overlaid on the T2w image (**a**, **h**). Loose matrix = *white*, calcification = *orange*, lumen = *red*, outer wall = *green*. Compared with the intensity of SCM muscle, regions with a hypointense signal on all four weightings are considered calcified tissue; while loose matrix (*white arrow*) is hyperintense on T2w and PDw image. As can be seen, calcification (*orange region*) could be detected with good repeatability in repeated scans by both segmentation methods (**f**, **m**, **g**, **n**); while loose matrix (*white regions*) could be detected with better agreement of size and location in two sessions by using automatic classification (**g**, **n**)

**Fig. 6:**
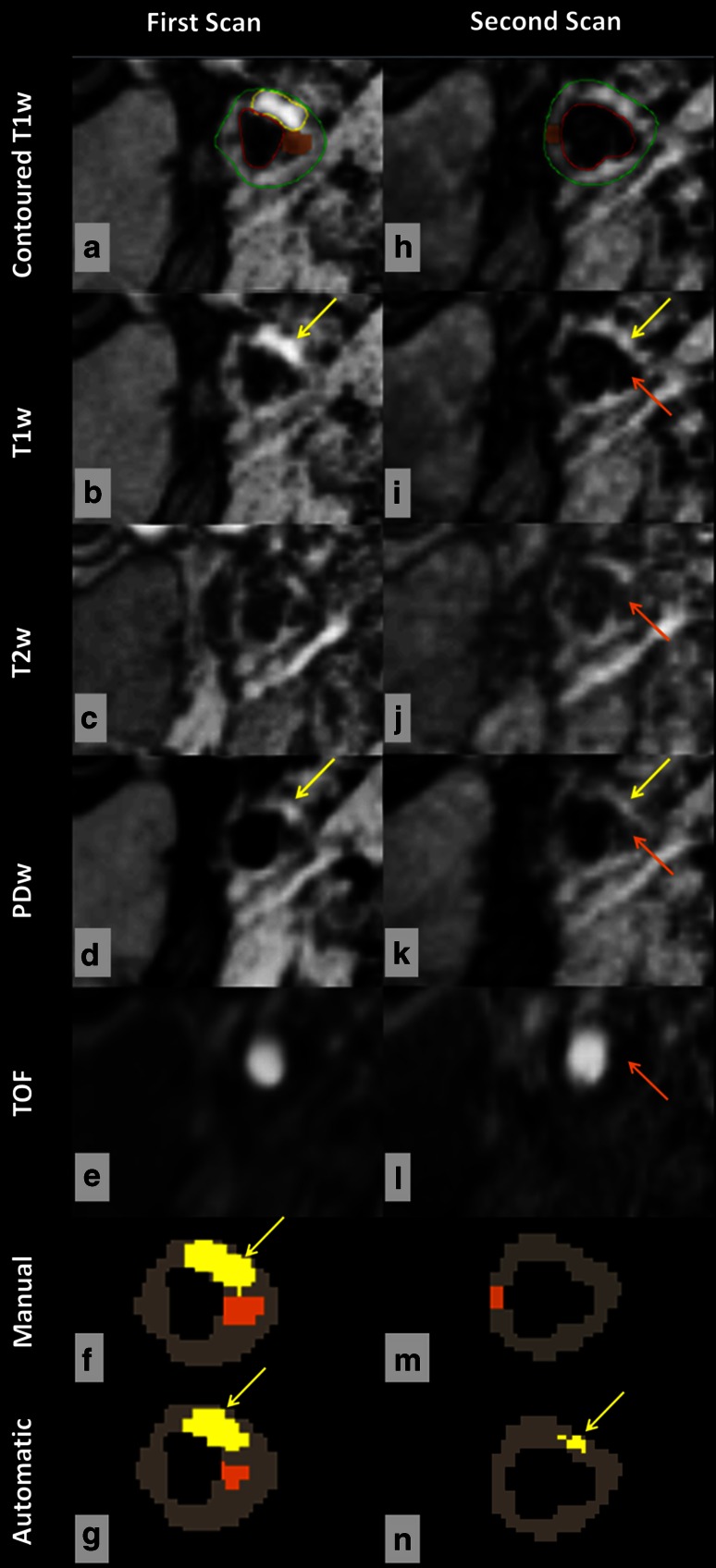
Manual and automated classification of lipid and calcification using multicontrast MR images obtained in repeated scans. Manual contours overlaid on the T1w image (**a**, **h**). Lipid = *yellow*, calcification = *orange*, lumen = *red*, outer wall = *green*. Using SCM muscle as reference, lipid (*yellow arrow*) appears hyperintense on T1w and PDw images. As can be seen, the classifier consistently identifies lipid (*yellow region*) in two scanning sessions (**g**, **n**), while the observer does not (**f**, **m**). Both segmentation methods could identify calcification (*orange region*) in the first scan (**f**, **g**), but no calcification was found at the same location in the second scan (**m**, **n**) because the observer annotated the region of juxta-luminal calcification (*orange arrow*) as lumen

Figures 5 and 6 show typical examples of manual and automated segmentation results for an aligned location at the first scan and second scan. As can be seen from these cases, the observer identified loose matrix (white region) and lipid (yellow region) in the first scan but not in the second scan, while the classifier reproducibly identified these two plaque components in both scanning sessions. The result from the classifier showed higher inter-scan repeatability of segmentation for lipid and loose matrix compared to the result from the observer.

## Discussion

In this study, we assessed the inter-scan repeatability of automated classification for in vivo quantification of plaque components in the carotid vessel wall from multi-contrast MRI. To the best of our knowledge, this is the first study that compares the scan–rescan repeatability of plaque composition quantification between manual and automated segmentation methods based on 3T MRI data. Our findings show that the presented automated segmentation approach demonstrates a significantly higher repeatability for plaque component area measurements compared to the manual segmentation procedure.

The goal of the development of automated segmentation tools is to make the segmentation of MR images more efficient, objective and repeatable. Our study was designed to validate if this goal is achievable in carotid vessel wall MR imaging. For calcification, the measurement repeatability was not significantly different between manual and automated methods. This might be explained by the fact that calcification appears relatively dark on all four contrast weightings, making the visual detection and manual segmentation for this component much easier. For lipid and loose matrix, which are more difficult to segment manually due to a larger intensity overlap with fibrous tissue in MR images, we found that the measurement repeatability was significantly improved by using automated classification. This observation confirmed that the automated classifier was more robust to the variations between scan–rescan images.

We assumed that because of the short time interval between the two scans of each patient, no pathological changes occurred between scan sessions. Nevertheless, variation in component area measurements between repeated scans can still be expected since the repeatability of area quantification is influenced by the repeatability of image acquisition. Variations in patient movement, patient position, the angle between the plaque and the MR slice between two scan sessions are unavoidable; therefore, in practice it is not possible to obtain perfect alignment at repeat scan sessions. Errors in the alignment may result in less overlap for a given plaque component between two time points, which leads to measurement variation. Compared to 2D acquisition, 3D acquisition, which uses thinner slices thickness and isotropic spatial resolution, could potentially reduce alignment error. Note that besides the image misalignment, additional sources of variability, including changes in magnetic field drift, prescan and shim setting, blood suppression and motion artifacts, make it infeasible to obtain identical images in repeated MR scanning of the same patient even though the same protocol and scanner are used. However, both manual and automated plaque assessment are similarly influenced by the scan–rescan variation. Changes in the appearance of the atherosclerotic vessel wall between inter-scan images in terms of plaque intensity impose challenges in the repeatable annotation of plaque components at a slice level based on visual inspection, since the human observer makes the decision for a region of interest to be a certain tissue based on its appearance, such as being dark, bright, thin or thick on MRI. In addition, the presence of particularly bright or dark regions might attract the observer’s bias towards a certain contrast weighting when making the decision. Instead, the automatic classification based on strictly normalized intensity features is objective and expected to be less sensitive to inter-scan variation of image appearance. Variations in the manual delineation of the lumen and outer wall contours between two time points will influence the repeatability of automatic plaque classification, as the classification is performed within the manually defined vessel wall region. However, based on the current result of high agreement between inter-scan carotid wall areas (scan: 62 ± 42 mm^2^ vs. rescan: 60 ± 38 mm^2^), we can safely conclude that manual vessel wall segmentation was consistent, and therefore it was not a major contributor to the inter-scan error of plaque classification.

In this study, we also assessed the automated-manual classification agreement of the classifier. As seen in Fig. 1, the classifier and the observer were comparable in classifying calcification. For lipid and loose matrix, Fig. 1 shows that the agreement between the automated and manual segmentation was significantly lower than the agreement between the repeated manual delineations. However, Fig. 3 and the result of Levene’s test shows that the automated classification was significantly more repeatable than the manual segmentation while analyzing the scan–rescan image data. These observations suggest that the automated classifier, which learned from the expert’s experience, on one hand strictly followed the classification rules based on the multi-contrast properties of the classes, and on the other hand, removed human error. In contrast, although the observer was repeatable in reanalyzing the same image, the repeatability of manual segmentation on the scan–rescan images was much lower. Furthermore, in longitudinal studies that aim at change detection, where the subjects can be used as their own references, scan–rescan repeatability might be of greater importance than automated-manual agreement. Therefore, automated analysis is a more reliable method for plaque components assessment in longitudinal studies.

This study has several limitations. First, no histology information was available to train and evaluate the automated classifier, as the patients were not scheduled for endarterectomy. Thus, the performance of the supervised classification is compromised by the limited accuracy of the manual segmentation. However, we used the well-documented manual plaque segmentation criteria that have been validated against histology [2]. More importantly, the patients in our cohort are those for whom the benefit of endarterectomy remains controversial and for whom non-surgical therapies remain possible [24, 25]. Second, a second observer was not available for performing plaque segmentation and assessing inter-observer variability in the current study. To train the classifier to be as reliable as possible, we used the plaque segmentation results from a well-trained observer, who is capable of analyzing plaque MRI data with comparable intra-observer agreement as in previous reports [2, 6]. Moreover, from literature [6], we expect the intra-observer variability is lower than the inter-observer variability; therefore, we can use the intra-observer variability to interpret the automated-manual variability, and thus a second observer is not indispensable. Third, in our patient population, the number of cases with hemorrhage was insufficient for automatic classification, as there was only one patient identified with hemorrhage. However, this study was designed to evaluate the repeatability of automatic plaque segmentation in a high-risk, yet stable, patient population, in which the prevalence of hemorrhage is low. Note that similar observation was found in previous studies. In a study by Biasiolli et al. [26], recent intra-plaque hemorrhage was found in one out of 15 patients; in the work of Li et al. [8], hemorrhage was detected only in two cases among 18 patients. Considering the clinical importance of intra-plaque hemorrhage, future studies need to be designed to evaluate the repeatability of automated classifiers for the detection of hemorrhage. Fourth, we used manual rigid alignment by shifting the image stack in the through-plane direction to obtain inter-scan registration, which may not be sufficient to correct for through-plane patient motion that is smaller than the slice thickness, or to correct for non-rigid patient motion between two different time points. 3D deformable registration could potentially improve registration (alignment) between two acquisitions, and therefore needs to be considered in future longitudinal studies. Finally, the inter-scan segmentation repeatability of our observer was slightly lower compared to a previous study [8]. This is probably due to the relatively advanced disease of our patients. As can be seen clearly in the baseline dimension characteristics of the tissue components from the current study, our patients have relatively high plaque volumes compared to those in other carotid MRI repeatability studies. The lower repeatability of the manual segmentation in our study might be explained by the fact that an increased plaque volume coincides with a more complex plaque phenotype with a higher number of different plaque components per patient [27]. In addition, moderate intra-observer scan–rescan repeatability is common in daily clinical practice. In spite of this, our automated classifier achieved satisfactory to good inter-scan segmentation repeatability for all major plaque components. Furthermore, our repeatability was based on area measurements, while in the previous study, the repeatability was based on volume measurements [8], which tend to increase the score because of the averaging effect of multiple slices.

## Conclusions

In conclusion, this study demonstrates that the scan–rescan repeatability of clinical prognostic plaque component quantification is significantly improved by using an automated classification approach. Therefore, automated atherosclerotic plaque classification, which provides complementary information to anatomical and morphological data, is a promising technique for treatment strategy evaluation and monitoring of disease progression.
